# Identification and characterization of *Clonorchis sinensis* cathepsin B proteases in the pathogenesis of clonorchiasis

**DOI:** 10.1186/s13071-015-1248-9

**Published:** 2015-12-21

**Authors:** Wenjun Chen, Dan Ning, Xiaoyun Wang, Tingjin Chen, Xiaoli Lv, Jiufeng Sun, De Wu, Yan Huang, Jin Xu, Xinbing Yu

**Affiliations:** Department of Parasitology, Zhongshan School of Medicine, Sun Yat-sen University, Guangzhou, Guangdong 510080 People’s Republic of China; Key Laboratory for Tropical Diseases Control of Ministry of Education, Sun Yat-sen University, Guangzhou, Guangdong 510080 People’s Republic of China; Guangdong Provincial Institute of Public Health, Guangdong Provincial Center for Disease Control and Prevention, Guangzhou, Guangdong 511430 China; Department of Medical Laboratory and Research Center, Tangdu Hospital, Fourth Military Medical University, Xi’an, Shaanxi 710038 China

**Keywords:** *Clonorchis sinensis*, Clonorchiasis, Cathepsin B, Pathogenesis

## Abstract

**Background:**

Human clonorchiasis is a prevailing food-borne disease caused by *Clonorchis sinensis* infection. Functional characterizations of key molecules from *C. sinensis* could facilitate the intervention of *C. sinensis* associated diseases.

**Methods:**

In this study, immunolocalization of *C. sinensis* cathepsin B proteases (*Cs*CBs) in *C. sinensis* worms was investigated. Four *Cs*CBs were expressed in *Pichia pastoris* yeast cells. Purified y*Cs*CBs were measured for enzymatic and hydrolase activities in the presence of various host proteins. Cell proliferation, wound-healing and transwell assays were performed to show the effect of *Cs*CBs on human cells.

**Results:**

*Cs*CBs were localized in the excretory vesicle, oral sucker and intestinal tract of *C. sinensis*. Recombinant y*Cs*CBs from yeast showed active enzymatic activity at pH 5.0–5.5 and at 37–42 °C. y*Cs*CBs can degrade various host proteins including human serum albumin, human fibronectin, human hemoglobin and human IgG. *Cs*CBs were detected in liver tissues of mice and cancer patients afflicted with clonorchiasis. Various bioassays collectively demonstrated that *Cs*CBs could promote cell proliferation, migration and invasion of human cancer cells.

**Conclusion:**

Our results demonstrated that *Cs*CBs can degrade various human proteins and we proved that the secreted *Cs*CBs are involved in the pathogenesis of clonorchiasis.

**Electronic supplementary material:**

The online version of this article (doi:10.1186/s13071-015-1248-9) contains supplementary material, which is available to authorized users.

## Background

Clonorchiasis is a food-borne parasitic disease caused by infection with *Clonorchis sinensis* (*C. sinensis*). Mammals are often infected with *C. sinensis* by consuming raw or uncooked fish or shrimp containing infectious metacercaria. Adult worms reside in the bile ducts of hosts and secreted products from *C. sinensis* eventually lead to clonorchiasis resulting in: cholangectasis, cholecystitis, cholelithiasis, hepatic fibrosis, and even liver cancer and bile duct cancer [1–3]. It is estimated that about 35 million people are afflicted with clonorchiasis, with most cases in Asian countries such as Korea, China and Vietnam [4, 5]. Food security problems caused by liver flukes have attracted high attention of public health, increasing the urgency of finding new approaches to prevent the spread of clonorchiasis. Clonorchiasis is listed among food-borne parasitic diseases requiring urgent control in China.

With the recent progress of the *C. sinensis* genome and transcriptome [6, 7], scientific researchers have expended much effort to elucidate the underlying mechanism of carcinogenic liver fluke associated hepatobiliary diseases [8, 9]. Molecular characterizations of key pathogenic molecules could speed up the interventions of *C. sinensis* infection. Cysteine proteases of helminthes have been widely characterized for their biological functions, including digestion, encystation, excystation, immune evasion and tissue invasion [10, 11]. Although cysteine proteases are abundant in *C. sinensis* transcriptome, limited information is available to illustrate the biological roles for *C. sinensis* in the host. Biological roles of *C. sinensis* cathepsin B proteases (*Cs*CBs) have not been sufficiently investigated, although extensive studies demonstrate the importance of cathepsins in other organisms.

In our previous work [12], we performed preliminary functional characterizations of four *C. sinensis* cathepsin B cysteine proteases (*Cs*CB1, *Cs*CB2, *Cs*CB3 and *Cs*CB4). *Cs*CBs were cloned into a prokaryotic expression vector (pET-28a) and expressed in the form of inclusion bodies in *E. coli*. Purified proteins from *E. coli* (e*Cs*CBs) were identified as *C. sinensis* excretory/secretory products and could trigger immune responses. However, we failed to perform further functional characterizations of these cysteine proteases due to loss of enzyme activity during the renaturation procedure. In this study, the eukaryotic expressing system in yeast was constructed using homologous recombination to express four y*Cs*CBs (*Cs*CB1, *Cs*CB2, *Cs*CB3 and *Cs*CB4) in *Pichia pastoris* X33 yeast cells. Recombinant y*Cs*CBs showed enzymatic activities and hydrolase activities in degrading various host proteins. *Cs*CB was detected in the liver tissues of mice and cancer patients afflicted with clonorchiasis. Recombinant *Cs*CB could promote cell proliferation, migration and invasion of human cancer cells. Our results provide evidence to support the role of *Cs*CBs in the pathogenesis of clonorchiasis.

## Methods

### Parasites, animals and patient samples

*C. sinensis* worms (larva, juvenile and adult) were freshly isolated from artificially infected freshwater fishes or Sprague–Dawley rats as we previously described [13]. Ethical Approval: Male Sprague–Dawley rats were purchased from the animal center at Sun Yat-sen University and raised in accordance with the National Institutes of Health animal care and ethical guidelines. BALB/c mice (8-weeks-old) were intragastrically infected with metacercariae to establish the *C. sinensis* infected mice model. Mice in the control group were treated with phosphate-buffered saline (PBS). The mice were sacrificed at 8 weeks after the infection and liver tissues were isolated for immunohistochemistry. Clonorchiasis-induced liver cancer specimens acquired from People’s Hospital of HengXian, Guangxi Zhuang Autonomous Region were pathologically diagnosed. Normal liver specimens were acquired from the first affiliated hospital of Sun Yat-Sen University. Ethical approval to use patients’ samples in this study was obtained from local hospitals and animal procedures were approved by the animal care and use committee of Sun Yat-sen University (Permit Numbers: SCXK (Guangdong) 2009–0011).

### Immunolocalization of *Cs*CBs in adult worm, cercaria and metacercaria

*C. sinensis* worms (larva, juvenile and adult) were used for the immunolocalization assay. Sectioned worms in paraffin wax were deparaffinized and incubated with previously prepared anti-*Cs*CBs sera (1: 400 in dilution). Pre-immune rat serum was applied as a negative control. Subsequently, the sections were incubated with Cy3 conjugated goat anti-rat IgG secondary antibody (1: 400 in dilution, Alexa Fluor 594, Molecular Probe) at RT for 1 h and imaged using an Axio Imager Z1fluorescent microscope (ZEISS).

### Homologous recombination of *Cs*CBs in yeast

As we previously reported, the complete coding sequences of *Cs*CBs range from 1014 to 1044 bp, with an N-terminal hydrophobic signal peptide ranging from 18 to 22 aa. To obtain recombinant *Cs*CBs from the eukaryotic expressing system for functional characterizations, we performed a homologous recombination of *Cs*CBs in the *Pichia pastoris* X33 yeast strain. The gene fragments of *Cs*CBs were amplified by PCR using primers (Table 1) according to *Cs*CBs-ORF (signal peptide excluded) and restriction sites of shuttle vector pPICZαB. Recombinant colonies were screened by Zeocin followed by validation using PCR and sequencing. Confirmed plasmids were extracted from DH5α and *Pichia pastoris* X33 was transformed with a *Sac* I linearized recombinant pPICZαB vector. The transformants were selected for Zeocin resistance on YPD plates [14]. Genomic DNAs were extracted from positive transformants for PCR to further confirm homologous recombination.

**Table 1:** Primers used in this study

Gene	Primers	Gene length
*Cs*CB1	F: 5′GCCGAATTCACGAGTATATTCCATCTTTC3′	960 bp
	R: 5′CGCTCTAGAAGCAGTTTTGGATGACCAG3′	
*Cs*CB2	F: 5′GTCGAATTCACGAAAATCTGGGGAGCGT3′	966 bp
	R: 5′GCCTCTAGAACAAAATGCGGAATGGTGG3′	
*Cs*CB3	F: 5′CGACTGCAGGAACAGAATCGATTGGACT3′	960 bp
	R: 5′GGCTCTAGAATCTTAAGTGGGATGCTGG3′	
*Cs*CB4	F: 5′GTACTGCAGGAAAACCAAAGCACGAAGC3′	987 bp
	R: 5′GCGGTCTAGAGGCGAAAAGGATTCATGATT3′	

### Expression and purification of y*Cs*CBs

Selected transformants were cultured in a BMGY medium for 16–18 h until OD_600_ of 2–6, cells were harvested by centrifugation and re-suspended in a BMMY medium at an OD_600_ of 1.0. The expression of y*Cs*CBs was induced by the daily addition of 0.5 % (*v*/*v*) methanol at 24, 48, 72 and 96 h, respectively. The culture filtrate of recombinant X33 cells was concentrated using ammonium sulfate. Concentrated supernatant was used for SDS-PAGE and Western blotting experiments to examine the extracellular expression of y*Cs*CBs in yeast. After that, recombinant protein was induced for 96 h and purified by His-bind resin chromatography (Novagen) followed by dialysis in PBS (pH7.2). Protein concentration was determined using the BCA method and stored at −80 °C for enzymatic assays.

### SDS-PAGE and Western blotting

Concentrated supernatant was subjected to 12 % SDS-PAGE stained with Coomassie brilliant blue. To further confirm extracellular expression of *Cs*CBs in X33 cells, concentrated supernatant was also subjected to Western Blotting. Protein samples were transferred onto a PVDF membrane (Millipore) followed by incubation with different antibodies: mouse anti-His antibody (1: 500 in dilution, Life Technologies), mouse anti-c-Myc monoclonal antibody (1: 500 in dilution, Life Technologies) and rat anti-*Cs*CB1 antibody (1: 800 in dilution), which was produced in our previous study. HRP-conjugated goat anti-mouse IgG or goat anti-rat IgG (1: 2,000 in dilution) was further incubated with each membrane, followed by enhanced chemiluminescence (ECL).

### Enzyme activity assays

The enzyme activity of y*Cs*CBs was assayed fluorometrically according to the previous report [15]. Enzyme reactions were performed under different enzyme concentrations, pH values and temperatures, respectively. Typically, the measurements were performed at 37 °C for 1 h in a 100 μl mixture containing y*Cs*CBs (0–20 μM), fluorescent Z-Phe-Arg-AMC/Z-Arg-Arg-AMC (20 μM, Bachem), 10 mM DTT, 0.05 % Brij-35 (AMRESCO), EDTANa_2_ (1 mM), and C_2_H_3_NaO_2_/Na_3_PO_4_/Tris–HCl (100 mM). The enzyme reaction was terminated by adding stop buffer (70 mM C_2_H_4_O_2_, 30 mM C_2_H_3_NaO_2_, 100 mM C_2_H_2_ClO_2_Na, pH 4.3). Fluorescent intensity was measured by plate reader at 348 nm.

### Degradation of host proteins

We first investigated hydrolase activity of y*Cs*CBs. Purified *Cs*CBs from *E. coli* or from *Pichia pastoris* were loaded into a 12 % SDS-PAGE containing 0.1 % gelatin. The gel was washed with washing buffer (2.5 % Tritonx-100, 50 mM Tris–HCl, 5 mM CaCl_2_, pH 7.5), followed by incubation with Na_3_PO_4_ (100 mM, pH 7.5) at 37 °C for 24 h. The hydrolase activity of y*Cs*CBs was visualized by Coomassie brilliant blue staining.

Second, we tested whether y*Cs*CBs could degrade host proteins, given that *Cs*CBs were proven components of secreted products of *C. sinensis* [12]. Purified y*Cs*CBs were incubated with host proteins at 37 °C for 2 h. Human serum albumin (MB-CHEM), human hemoglobin (MB-CHEM), human IgG (MB-CHEM), human fibronectin (Sigma) and bovine serum albumin (MB-CHEM) were used as the substrates. The assays were performed in a 200 μl mixture containing Na_3_PO_4_ (100 mM, pH 5.5), EDTANa_2_ (1 mM), DTT (10 mM), y*Cs*CBs (20 μM) and various host proteins (1 mg). The reactions were terminated by adding a reducing sample buffer and analyzed by SDS-PAGE.

### Inhibition effect on enzyme activity of y*Cs*CBs

To confirm the specificity of enzyme activity from the above-mentioned assays, we performed enzymatic inhibition experiments by using different enzyme inhibitors purchased from Sigma. Briefly, y*Cs*CBs (20 μM) were pre-incubated with or without protease inhibitors, including E-64 (20 μM), iodoacetic acid (10 μM), PMSF (2 mM), EDTA (2 mM), AEBSF (200 μM), TPCK (200 μM) and CA-074 methyl ester (1 μM). Z-Phe-Arg-AMC (20 μM) was added to the reactions after 30 min and incubated for another 1 h. Each assay was performed in triplicate and enzyme activity was measured by plate reader at 348 nm.

### Immunohistochemistry of *Cs*CB in infected mouse and patient

Next, we sought to investigate whether *Cs*CBs are involved in the pathogenesis of clonorchiasis using the y*Cs*CB4 protein. First, we performed an immunohistochemistry using an anti-*Cs*CB4 antibody to see the localization of *Cs*CB4 in liver tissues of clonorchiasis afflicting mice and patients. Generally, tissue samples were fixed in 10 % formalin and sectioned to 4 μm in thickness. The sections were routinely treated with ethanol and slides were immersed in a 0.3 % hydrogen peroxide solution for 20 min to block the endogenous peroxidase activity. The sections were then incubated overnight at 4 °C with rat anti-*Cs*CB4 antibody (1: 100 in dilution). Sections were subsequently incubated with horseradish peroxidase (HRP) conjugated rat-specific secondary antibodies (1: 200 in dilution). Immunohistochemistry results were developed using diaminobenzidine (DAB) and counterstained with hematoxylin. The images were taken under a light microscope (Leica DMI3000B) and subsequently analyzed using ImagePro Plus software (Media Cybernetics, Roper, USA). The brown staining was indicated as Integrated Optical Density (IOD), and IOD/Area was indicated as a relative expression level of *Cs*CB4 in liver tissues.

### Cell proliferation analysis

The cell proliferation level induced by y*Cs*CB4 was measured in two human cancer cell lines, human hepatocellular carcinoma cell line (MHCC-97H, ATCC) and human cholangiocarcinoma cell line (RBE, ATCC). MHCC-97H and RBE cells were grown in DMEM (Hyclone, USA) and RPMI-1640 (Gibco, USA), respectively and supplemented with 10 % fetal bovine serum (Gibco, USA) and 1 % penicillin/streptomycin (Gibco, USA). Cells were incubated at 37 °C in a humidified chamber under 5 % CO_2_. Cells at the logarithmic phase were plated into 96-well plates in triplicate and treated with y*Cs*CB4 protein (1 μg/ml). Cell viability at 24 h was measured using Cell Counting Kit-8 (CCK-8) as previously described [16]. For cell cycle analysis using flow cytometry, cells were incubated with y*Cs*CB4 (1 μg/ml) for 24 h. Then the cells were trypsinized and fixed in 100 % ethanol at −20 °C overnight. Cell cycle distribution was determined by fluorescence activated cell sorting (FACS). Data was analyzed using the FlowJo software.

### Cell migration and invasion assay

To further confirm the role of y*Cs*CB4 in human cancer cell growth, wound-healing assays were performed to evaluate the effect of y*Cs*CB4 on cell migration according to the previous method [17]. MHCC-97H and RBE cells seeded in 6-well plates were grown to 80 % confluence and incubated with y*Cs*CB4 (1 μg/ml) or PBS for 24 h. Cells were wounded by scratching with pipette tips. Wounds at 24 h were observed and photographed under a light microscope (Leica DMI3000B).

To evaluate the effect of y*Cs*CB4 on cell invasion, we performed transwell assays according to the method described elsewhere [18]. MHCC-97H and RBE cells were suspended in serum-free media and placed in 8 μm pores. These inserts were placed in wells with serum-containing media. Cells were incubated with y*Cs*CB4 (1 μg/ml) or PBS for 24 h. Invasion assays were performed using matrigel-coated membranes (BD, USA). The migration assay was similar to the invasion assay, except that the upper side of the membranes was not coated with the matrigel. Cells attached to the lower surface of the membranes at 24 h were counted under a light microscope.

### Statistical analysis

Experimental data were obtained from three independent experiments with a similar pattern; data are expressed as means ± standard deviation. All the data were analyzed by SPSS 13.0. Student’s *t*-test and ANOVA were used to analyze the data. *P* value <0.05 was considered statistically significant.

## Results

### Immunolocalization of *Cs*CBs in *C. sinensis* worms

**Fig. 1:**
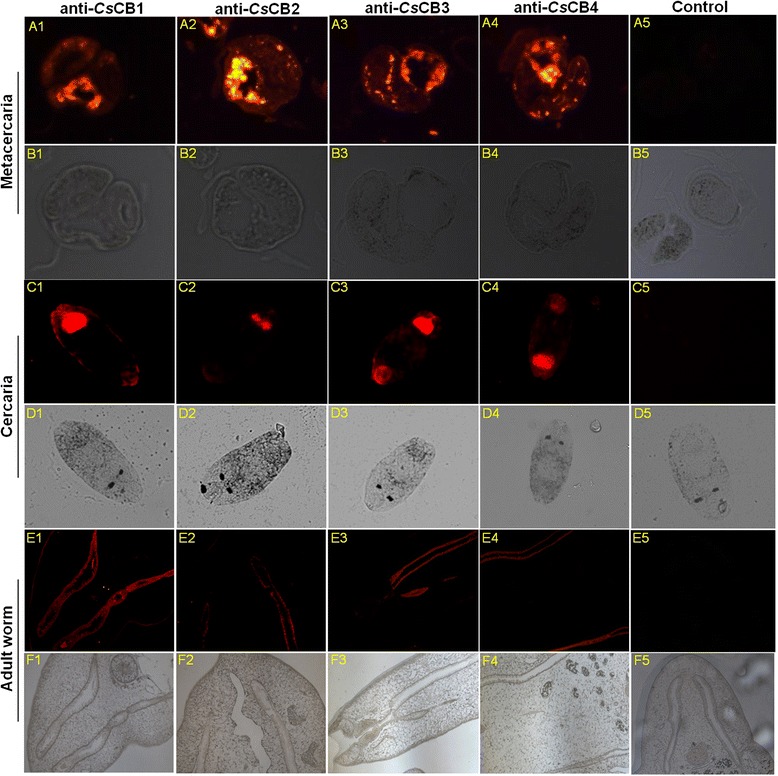
Immunolocalization of *Cs*CBs in *C. sinensis* worms. Sectioned worms were deparaffinized and incubated with anti-*Cs*CBs sera (1: 400). Pre-immune rat serum was applied as a negative control. The sections were incubated with Cy3 conjugated goat anti-rat IgG secondary antibody (1: 400) in dark and imaged using an Axio Imager Z1 fluorescent microscope. 1–5 indicated anti-*Cs*CB1 serum, anti-*Cs*CB2 serum, anti-*Cs*CB3 serum, anti-*Cs*CB4 serum and pre-immune serum, respectively. **a**, **b** Metacercaria. **c**, **d** Cercaria. **e**, **f** Adult worm. **a**, **c**, **e** Images under fluorescent objective. **b**, **d**, **f** Images in bright field

In our previous work, we demonstrated that *Cs*CBs are components of *C. sinensis* secreted products by Western Blotting assay [12]. In this study, we first investigated the immunolocalization of four *Cs*CBs in *C. sinensis* worms. As shown in Fig. 1, in metacercaria (Fig. 1, panel A1-A4) and cercaria (Fig. 1, panel C1-C4), four *Cs*CBs could be detected in the excretory vesicle and oral sucker. In adult worm, four *Cs*CBs could be specifically observed in the intestinal tract (Fig. 1, panel E1-E4). No fluorescent signal could be detected in negative controls treated with pre-immune serum (Fig. 1, panel A5, C5 and E5).

### Homologous recombination of *Cs*CBs in yeast

**Fig. 2:**
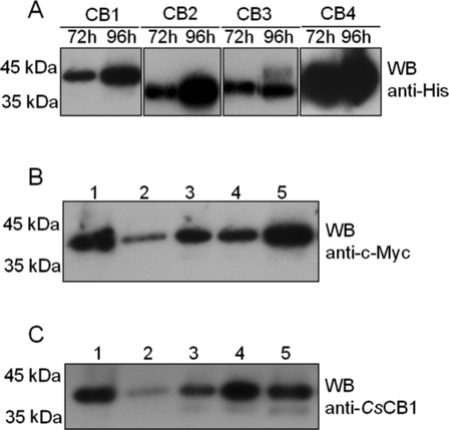
Identification of extracellular expression of y*Cs*CBs by Western Blotting. **a** Cell culture medium of y*Cs*CBs (y*Cs*CB1, y*Cs*CB2, y*Cs*CB3 and y*Cs*CB4) was probed with mouse anti-His antibody (1: 500). **b** Cell culture medium of y*Cs*CB1 was probed with mouse anti-c-Myc antibody (1: 500). **c** Cell culture medium of y*Cs*CB1 was probed with rat anti-*Cs*CB1 antibody (1: 800). Lanes 1–5 indicated ammonium sulfate precipitate, 72-h culture medium, 96-h culture medium, 120-h culture medium and 144-h culture medium, respectively

As shown in Additional file 1: Figure S1, the ORFs of four *Cs*CBs were successfully inserted into the shuttle vector pPICZαB. Selected transformants with *Cs*CBs were used for protein expression induced by methanol. Cells were collected at different time points to monitor the expression level of y*Cs*CBs. As shown in Additional file 1: Figure S2, a band of interest around 45 kDa appeared in the yeast culture medium at 24 h with an increased expressed level afterward, indicating the successful expression of y*Cs*CBs in yeast (Additional file 1: Figure S2A-D). To further confirm whether the interesting band (45 kDa) was y*Cs*CBs, a concentrated culture medium was used for Western blotting assays probed with different antibodies (Fig. 2 and Additional file 1: Figure S3). As expected, the interesting bands seen in SDS-PAGE could be recognized by His-tag (Fig. 2a). Expression of y*Cs*CBs was also demonstrated by reactions with anti-c-Myc antibody and anti-*Cs*CB1 antibody when y*Cs*CB1 was used as the example (Fig. 2b and c). Eventually, four y*Cs*CBs were purified by His-bind resin chromatography and analyzed by 12 % SDS-PAGE, resulting in a highly pure y*Cs*CBs (Additional file 1: Figure S4, A-D).

### Enzyme activity of y*Cs*CB

**Fig. 3:**
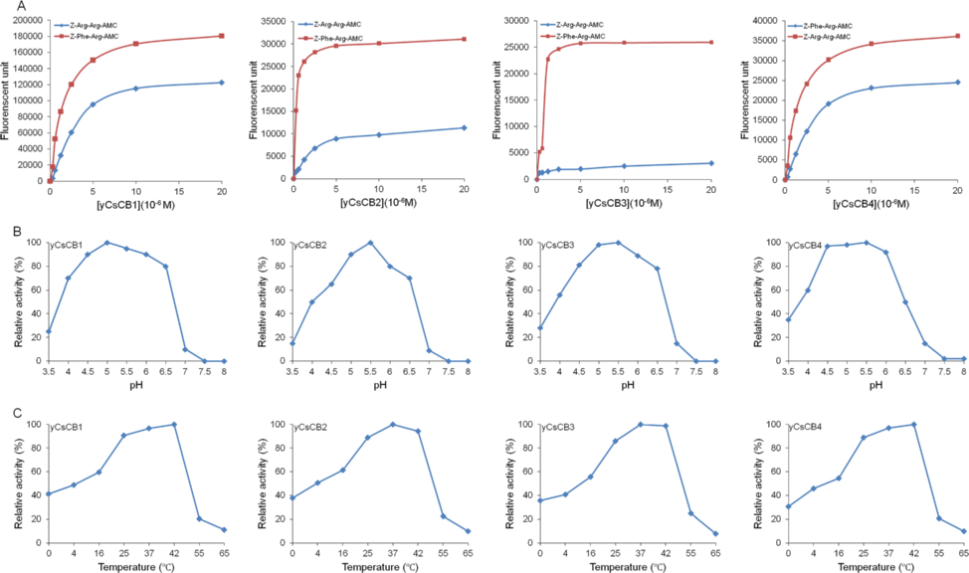
Enzyme activity of y*Cs*CB. **a** Enzyme activity of y*Cs*CBs was assayed by using two fluorescent substrates (Z-Phe-Arg-AMC and Z-Arg-Arg-AMC) under different enzyme concentrations (0, 0.3125, 0.625, 1.25, 2.5, 5, 10 and 20 μM). **b** Enzyme activity of y*Cs*CBs was assayed by using fluorescent Z-Phe-Arg-AMC as a substrate under different pH values (3.5, 4.0, 4.5, 5.0, 5.5, 6.0, 6.5, 7.0, 7.5 and 8.0). **c** Enzyme activity of y*Cs*CBs was assayed by using fluorescent Z-Phe-Arg-AMC as substrate under different temperatures (0, 4, 16, 28, 37, 42 and 55 °C). Fluorescent intensity was measured at 348 nm to calculate relative enzyme activity

We tested the enzyme activity of y*Cs*CBs by using two fluorescent substrates (Z-Phe-Arg-AMC and Z-Arg-Arg-AMC). As shown in Fig. 3a, four y*Cs*CBs were demonstrated to be active enzymes when the enzyme concentration ranged from 0 to 20 μM. Compared to Z-Arg-Arg-AMC, y*Cs*CBs showed higher enzymatic activity when Z-Phe-Arg-AMC was used as the substrate. In addition, optimal enzyme reaction pH values and temperatures were investigated. y*Cs*CBs showed the highest enzymatic activity when enzyme assays were performed at pH 5.0–5.5 (Fig. 3b) and at 37–42 °C (Fig. 3c). The results suggested that y*Cs*CBs were stable enzymes under acidic conditions when temperatures ranged from 37 to 42 °C.

### Host proteins degradation by y*Cs*CBs

**Fig. 4:**
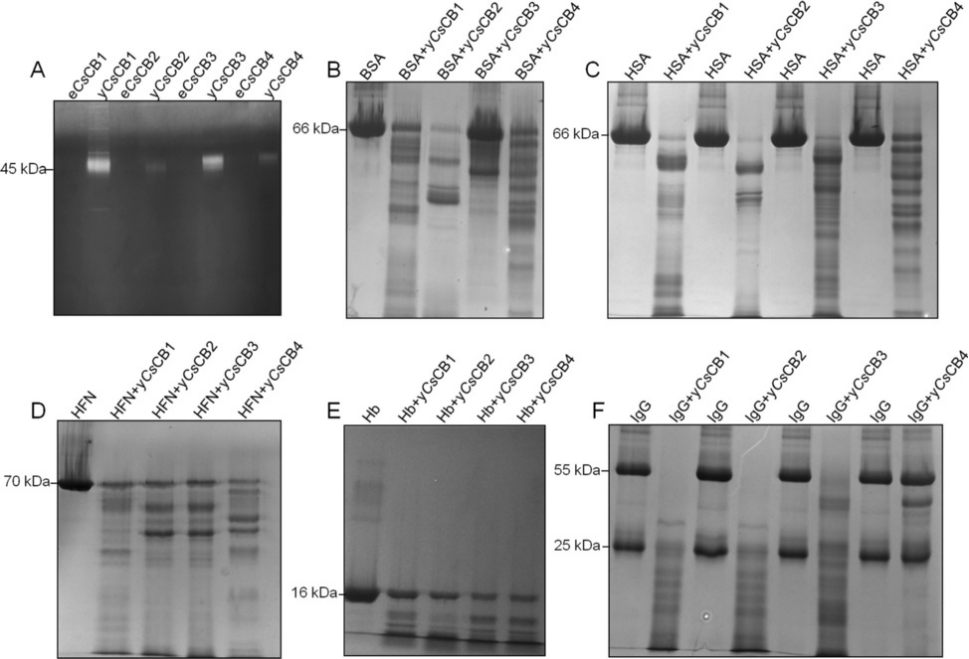
Host proteins degradation by y*Cs*CBs. The assays were performed in a 200 μl mixture containing Na_3_PO_4_ (100 mM, pH 5.5), EDTANa_2_ (1 mM), DTT (10 mM), y*Cs*CBs (20 μM) and various host proteins (1 mg). The reactions were terminated by adding a reducing sample buffer and analyzed by SDS-PAGE. **a** Gelatin hydrolysis assay using y*Cs*CBs versus e*Cs*CBs. **b** Degradation assay using bovine serum albumin (BSA) as the substrate. **c** Degradation assay using human serum albumin (HSA) as the substrate. **d** Degradation assay using human fibronectin (HFN) as the substrate. **e** Degradation assay using human hemoglobin (Hb) as the substrate. **f** Degradation assay using human IgG as the substrate. The molecular mass of host proteins was indicated

Since *Cs*CBs have been demonstrated to be the components of secreted products of *C. sinensis*, we tested hydrolase activity of y*Cs*CBs within the context of host proteins under acidic conditions (pH 5.5). In gelatin hydrolysis assay (Fig. 4a), y*Cs*CBs could obviously hydrolyze gelatin, while e*Cs*CBs could not. When bovine serum albumin was used as the substrate, four y*Cs*CBs also showed a degradation effect with different activities (Fig. 4b). We then employed different host proteins as the substrate; four y*Cs*CBs could degrade host proteins including human serum albumin (Fig. 4c), human fibronectin (Fig. 4d), human hemoglobin (Fig. 4e) and human IgG (Fig. 4f).

### Inhibition effect on enzyme activity of y*Cs*CBs

**Fig. 5:**
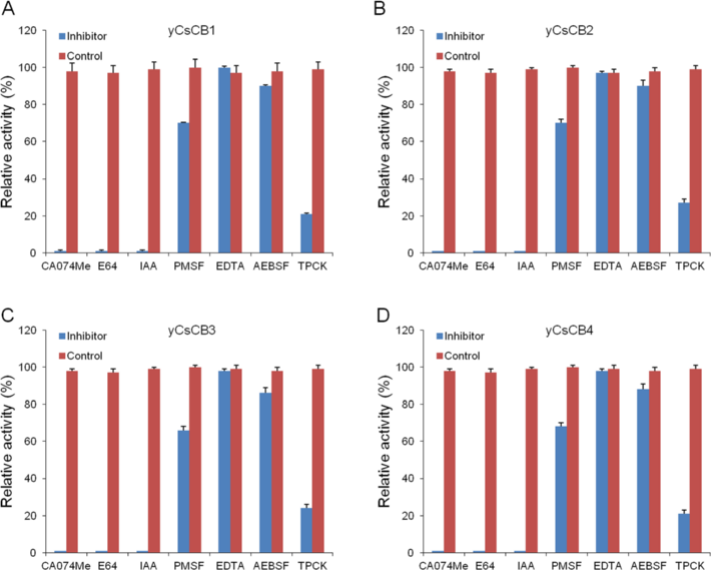
Inhibition effect on enzyme activity of y*Cs*CBs. **a**-**d** y*Cs*CB1, y*Cs*CB2, y*Cs*CB3 and y*Cs*CB4, respectively. y*Cs*CBs (20 μM) were pre-incubated with or without protease inhibitors including E-64 (20 μM), iodoacetic acid (10 μM), PMSF (2 mM), EDTA (2 mM), AEBSF (200 μM), TPCK (200 μM) and CA-074 methyl ester (1 μM). Z-Phe-Arg-AMC (20 μM) was added to the reactions after 30 min and incubated for another 1 h. Fluorescent intensity was measured at 348 nm to calculate relative enzyme activity. Errors represent data from triplicate samples

As we showed above, four y*Cs*CBs were demonstrated as active enzymes. We carried out inhibiting assays using different enzyme inhibitors to confirm that observed enzyme activity was specific to cathepsin B proteases (Fig. 5). Compared to controls without enzyme inhibitors, enzymatic activities of y*Cs*CBs could be completely inhibited by cathepsin B specific inhibitors or cysteine protease specific inhibitors (CA-074 methyl ester, E-64 and iodoacetic acid). However, serine protease specific inhibitors (PMSF and AEBSF) and trypsin specific inhibitor TPCK could only partially inhibit enzyme activity. EDTA had no inhibition on the activity, indicating that *Cs*CBs belongs to the typical cathepsin B cysteine protease family.

### Immunohistochemistry of *Cs*CB in infected mice and liver cancer patients

**Fig. 6:**
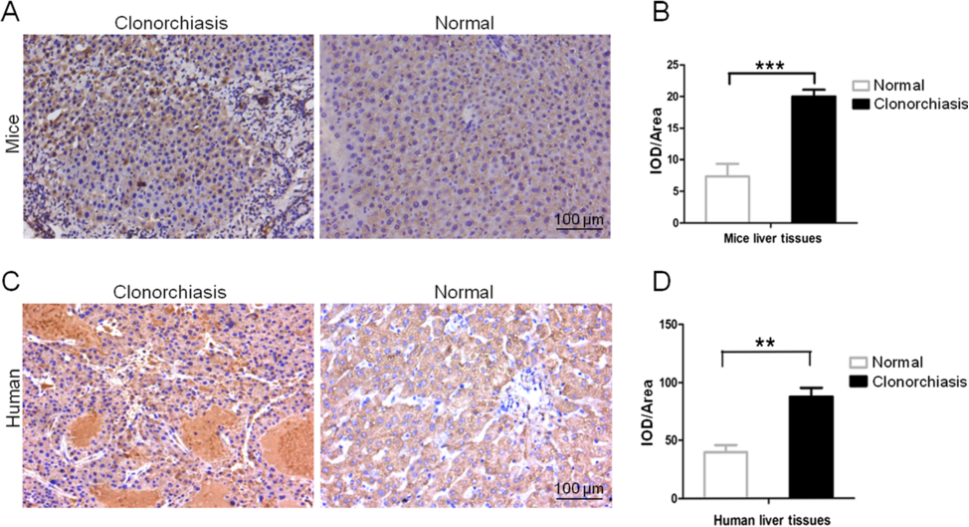
Immunohistochemistry assays of *Cs*CB4 in liver tissues. **a** Representative localization image of *Cs*CB4 in mice liver. **c** Representative localization image of *Cs*CB4 in human liver. **b**, **d** Quantification of Integrated Optical Density (IOD). The images were magnified at 200X and scale bar is 100 μm. The brown staining was indicated as IOD; IOD/Area was indicated as a relative expression level of *Cs*CB4 in liver tissues. Five random fields from each sample were analyzed using ImagePro Plus software. The sections were developed by DAB and counterstained with hematoxylin. **P < 0.01, ***P < 0.001, compared to normal tissue

The liver tissues from mice model and patient samples were analyzed by immunohistochemistry using rat anti-*Cs*CB4 antibody. Positive staining was indicated with brown. Compared to normal mice, strong staining was detected in the liver tissue of infected mice (Fig. 6a). The IOD of infected mice livers was significantly higher than the IOD of normal mice livers (Fig. 6b, *P* < 0.001). Strong staining dispersed throughout the liver tissues from clonorchiasis patients, while little brown staining was evident in liver tissues from healthy people (Fig. 6c). The IOD of liver cancer specimens was higher than the IOD of normal liver specimens (Fig. 6d, *P* < 0.01).

### Cell proliferation promoted by *Cs*CB4

**Fig. 7:**
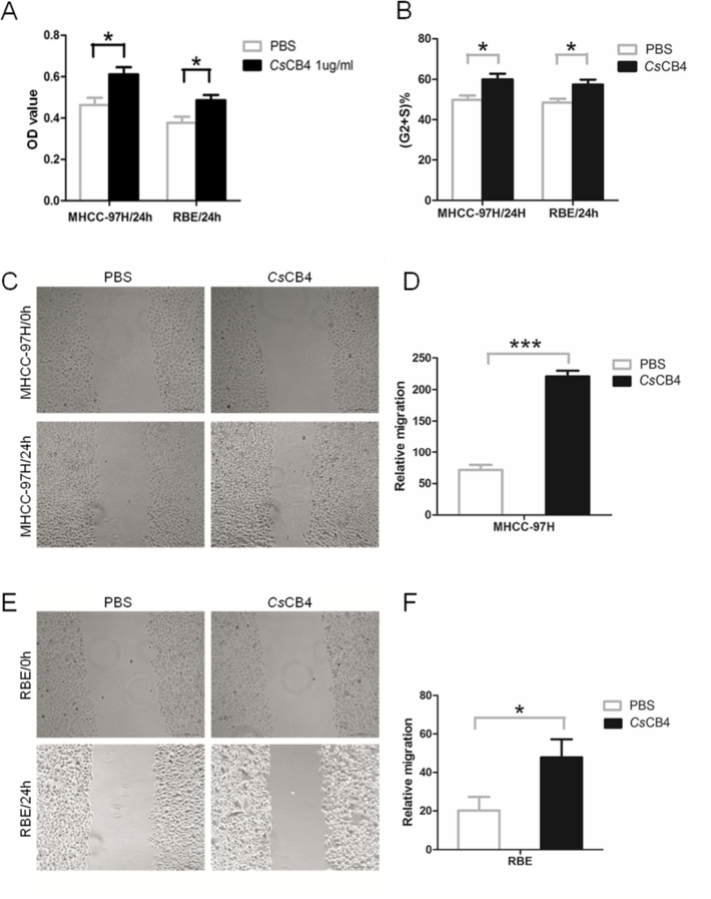
The effect of y*Cs*CB4 on human MHCC-97H and RBE cells. **a** Cell proliferation level measured by CCK-8 assay. **b** Cell cycle analysis by flow cytometry, the percentage of cells in the G2/S period was analyzed and quantified using FlowJo software. **c**, **d** Cell migration of MHCC-97H cells shown by wound-healing assay. Cells were observed using light microscope under 10X objective. **e**, **f** Cell migration of RBE cells shown by wound-healing assays; cells were observed using a light microscope under 10X objective. Generally, MHCC-97H cells or RBE cells were incubated with 1 μg/ml of y*Cs*CB4 or PBS for 24 h and assays were performed. Assays were performed in triplicate. Relative cell migration level was calculated by normalizing to cell migration level at 0 h. *P < 0.05, ***P < 0.001, compared to PBS control

The proliferation level of MHCC-97H and RBE cells treated with y*Cs*CB4 was measured by CCK-8 assays. As shown in Fig. 7a, both MHCC-97H and RBE cells treated with y*Cs*CB4 showed significantly a higher proliferative level when compared to control cells (*P* < 0.05). To further confirm the effect of y*Cs*CB4 on cell proliferation, we evaluated the distribution of the cell cycle by flow cytometry (Additional file 1: Figure S5). As shown in Fig. 7b, the G2/S percentage of MHCC-97H and RBE cells treated with y*Cs*CB4 was statistically higher than those of cells treated with PBS control, respectively (*P* < 0.05).

### Cell migration and invasion triggered by *Cs*CB4

**Fig. 8:**
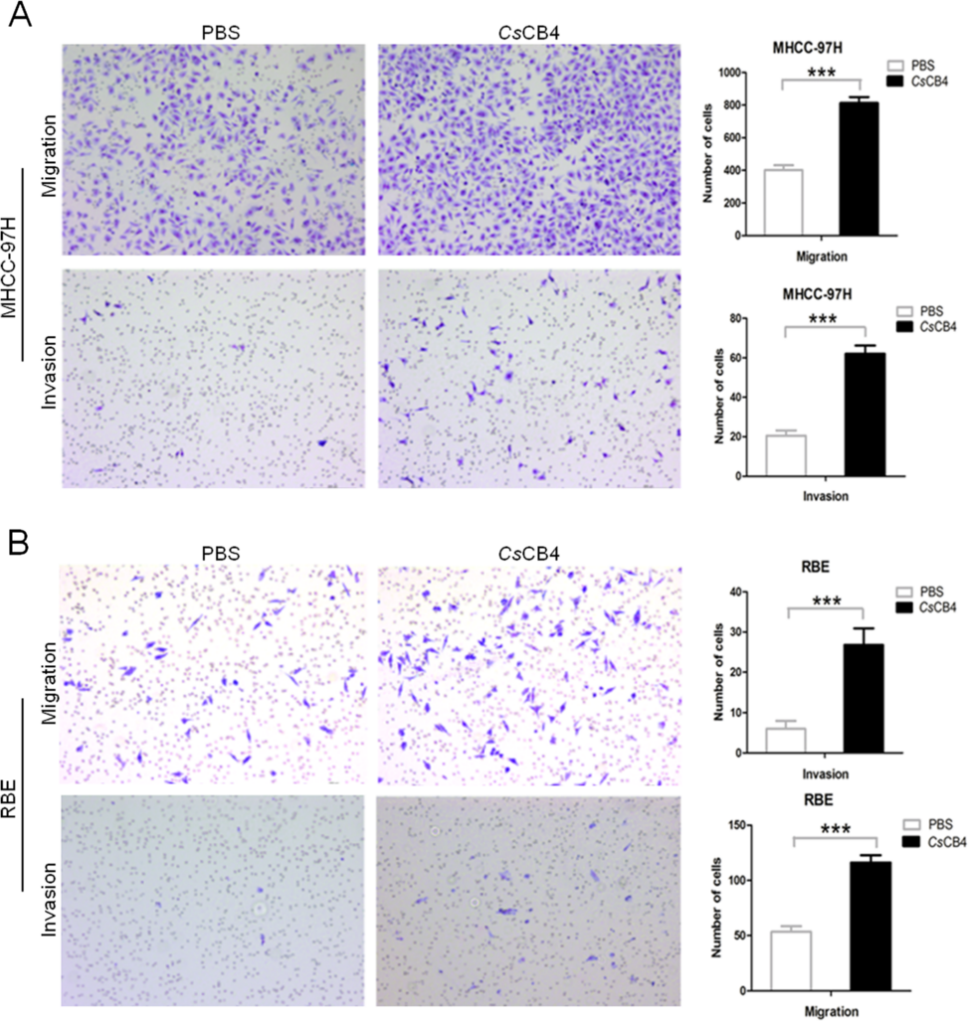
Cell migration and invasion triggered by *Cs*CB4 in transwell assay. MHCC-97H cells (**a**) or RBE cells (**b**) were suspended in serum-free media. Cells were incubated with 1 μg/ml of y*Cs*CB4 or PBS for 24 h. Invasion assays were performed using matrigel-coated membranes. Migration assay was similar to the invasion assay, except that the upper side of the membranes was not coated with the matrigel. Cells attached to the lower surface of the membranes at 24 h were counted under a light microscope. Ten random visual fields were selected to quantify the migration and invasion. ***P < 0.001, compared to PBS control

We wondered if y*Cs*CB4 could play any role in cancer cell migration. To determine this, we carried out wound-healing assays. For both MHCC-97H (Fig. 7c) and RBE cells (Fig. 7e), at the concentration of 1 μg/ml, y*Cs*CB4 could induce significant cell migration, which is 3-fold (Fig. 7d, *P* < 0.001) and 2-fold (Fig. 7f, *P* < 0.05) when compared to the PBS control, respectively. Similarly, in transwell assays, y*Cs*CB4 (1 μg/ml) promoted a higher cell migration level in MHCC-97H (Fig. 8a) and RBE cells (Fig. 8b). In addition, the cell invasion level could also be reflected in transwell assays, indicating that at the concentration of 1 μg/ml y*Cs*CB4 could induce a 3-fold (Fig. 8a, *P* < 0.001) and 5-fold (Fig. 8b, *P* < 0.001) invasion level compared to the PBS control, respectively.

## Discussion

Proteases are ubiquitous in nature and most organisms. In addition to their housekeeping functions, proteases are involved in the digestion of host proteins such as fibronectin, collagen and albumin, to facilitate migration and feeding in the host [19]. Cathepsins are of particular interest to parasitologists because there is considerable evidence that cathepsins are involved in parasitism. All of the trematodes have been shown to contain genes encoding cathepsin B-like proteins. For example, in *Fasciola hepatica*, cathepsin B was identified as an important factor associated with invasion of the mammalian host [20] and cathepsin B was suggested as a potential digestive factor in newly excysted juvenile parasites [21]. Cathepsin B was also identified as a stage and tissue-specific expression protease in *Fasciola gigantica* [22]. In *Schistosoma mansoni*, secreted cathepsin B was proposed to interact with host molecules and thus be a vital factor in parasitism [23]. In *Angiostrongylus cantonensis*, cathepsin B plays a potential role in the invasion of the central nervous system during parasite-host interactions [24]. Thus, cathepsin B proteases clearly play an important role in the biology of trematode parasites. Cysteine proteases were abundant genes in *C. sinensis* genome and transcriptome. As the main components of *C. sinensis* excretory/secretory products, *Cs*CBs were proved to be potential vaccine candidates and diagnostic markers [11, 12]. In this study, we constructed a eukaryotic expressing system by homologous recombination to express four *Cs*CBs in yeast. Active y*Cs*CBs were purified for biochemical and functional characterizations. The cellular effect of *Cs*CBs on human cancer cells was observed using various cellular assays. Our results provide evidence to support the role of *Cs*CBs in the pathogenesis of clonorchiasis.

At this time, *Cs*CBs were expressed in soluble form with enzyme activity because of the advantages of the methylotropic yeast, *Pichia pastoris*, and the shuttle vector pPICZαB [25, 26]. This shuttle vector facilitated our transformation operation from the *E. coli* system to the *Pichia pastoris* system. y*Cs*CBs showed active enzyme activity with a wide range of pHs, while peak enzymatic activity was assayed at pH 5.0–5.5, suggesting that y*Cs*CBs were functional enzymes under acidic conditions. The hypothesis that *Cs*CBs are acidic enzymes located in gut of flukes could be supported by previous reports [23, 27, 28], considering the fact that the pH of the gut lumen of *Fasciola hepatica* has been suggested to be pH 5.5 [29]. Enzymatic activities of y*Cs*CBs could be completely inhibited by cathepsin B specific inhibitors or cysteine protease specific inhibitors, while serine protease specific inhibitors and trypsin specific inhibitor showed only a weak inhibition effect. These results helped us confirm that our obtained y*Cs*CBs belong to the typical cathepsin B cysteine protease family. In addition to typical enzymatic activities, y*Cs*CBs could degrade all tested host proteins such as human serum albumin, human fibronectin, human hemoglobin and human IgG. Those host proteins have been used in other parasites to test the digestive effect of cathepsins [19]. For instance, *Fh*CB2 could cleave serum albumin and IgG, indicating a role in the digestion of protein substrates for nutritional purposes [30]. Recombinant *Fg*CB3 was recently shown to digest fibronectin, consistent with a role in digesting connective tissue and host invasion [31]. The digestive effect of y*Cs*CBs supports our hypothesis that *Cs*CBs serve as key virulence factors for *C. sinensis*, it is most likely that *Cs*CBs are involved in the pathogenesis of clonorchiasis. The biological role of *Cs*CB could be implied by immunolocalization results, showing that *Cs*CBs were localized in the excretory vesicle, oral sucker and intestinal tract of *C. sinensis* worms. Immunolocalization of *Cs*CB is similar to *C. sinensis* cathepsin F, which is also a secreted protein in the intestine of *C. sinensis* [32]. These two enzymes were expressed throughout developmental stages of the parasite. Given that *Cs*CBs and *Cs*CFs are from the same protease family, it is reasonable to assume they are synthesized in epithelial cells lining the parasite intestine followed by secretion into the intestinal lumen of the parasite, to play a role for nutrient uptake in the parasite [33–35].

As the key component of secreted products, many proteins have been connected with hepatobiliary diseases observed in individuals infected with liver flukes [36, 37]. It was suggested that secreted products released by liver flukes could lead to pathologic changes in biliary epithelial cells [38, 39]. Human cells exposed to ESPs from liver flukes (*C. sinensis*, *Fasciola hepatica*, and *Opisthorchis viverrini*) showed diverse pathophysiological responses including proliferation, apoptosis and inflammation [40–42]. In human diseases, experimental and clinical evidence have linked cathepsin B with tumor invasion and metastasis. Cathepsin B expression increases in many human cancers at mRNA, protein and activity levels [43]. In this study, we found that *Cs*CB4 was detected in liver tissues from infected mice or liver cancer patients induced by clonorchiasis. To gain a better understanding of *Cs*CBs-associated human diseases, we measured the biological effects of y*Cs*CB4 protein on human cancer cells. The results from different approaches demonstrated that y*Cs*CB4 could promote cell proliferation, cell migration and cell invasion of human hepatocellular carcinoma cells and human cholangiocarcinoma cells. Our observed results could be supported by our previous report that severin protein from *Cs*ESPs had an anti-apoptotic role in hepatocarcinoma PLC cells [44]. Given that four *Cs*ESPs have similar biochemical properties, it is conceivable that *Cs*CBs are involved in the pathogenesis of clonorchiasis during *C. sinensis* infection. However, further investigations are required in order to identify precise mechanisms to provide therapeutic strategies for clonorchiasis. With RNA interference applications in helminth [45, 46], it is feasible to perform a *Cs*CBs-mediated intervention in *C. sinensis* associated diseases.

## Conclusion

In summary, we expressed and purified four *Cs*CBs in yeast and demonstrated that *Cs*CBs can degrade various human proteins. *Cs*CBs could be detected at a high expression level in clonorchiasis-induced liver cancer tissues. In addition, our results indicate that *Cs*CBs could confer proliferative and invasive role in human cancer cells. The present study supports the involvement of *Cs*CBs in the pathogenesis of clonorchiasis.

## Additional file

Additional file 1: Figure S1. Identification of recombinant plasmids by PCR amplification and restriction enzyme digestion. **Figure S2.** Identification of extracellular expression of yCsCBs by 12 % SDS-PAGE. **Figure S3.** Identification of extracellular expression of yCsCBs by Western Blotting. **Figure S4.** Identification of purified protein of CsCBs by SDS-PAGE. **Figure S5**. Representative cell cycle analysis by flow cytometry. (DOCX 5346 kb)
