# Supplementary material for: Identification and characterization of Clonorchis sinensis cathepsin B proteases in the pathogenesis of clonorchiasis
Source: Parasit Vectors. 2015 Dec 21;8:647. doi: 10.1186/s13071-015-1248-9 (PMC4687107; doi:10.1186/s13071-015-1248-9)
Supplement: Additional file 1: Figure S1. — Identification of recombinant plasmids by PCR amplification and restriction enzyme digestion. Figure S2. Identification of extracellular expression of yCsCBs by 12 % SDS-PAGE. Figure S3. Identification of extracellular expression of yCsCBs by Western Blotting. Figure S4. Identification of purified protein of CsCBs by SDS-PAGE. Figure S5. Representative cell cycle analysis by flow cytometry. (DOCX 5346 kb) [file 13071_2015_1248_MOESM1_ESM.docx]

**Additional files:**

Figure S1


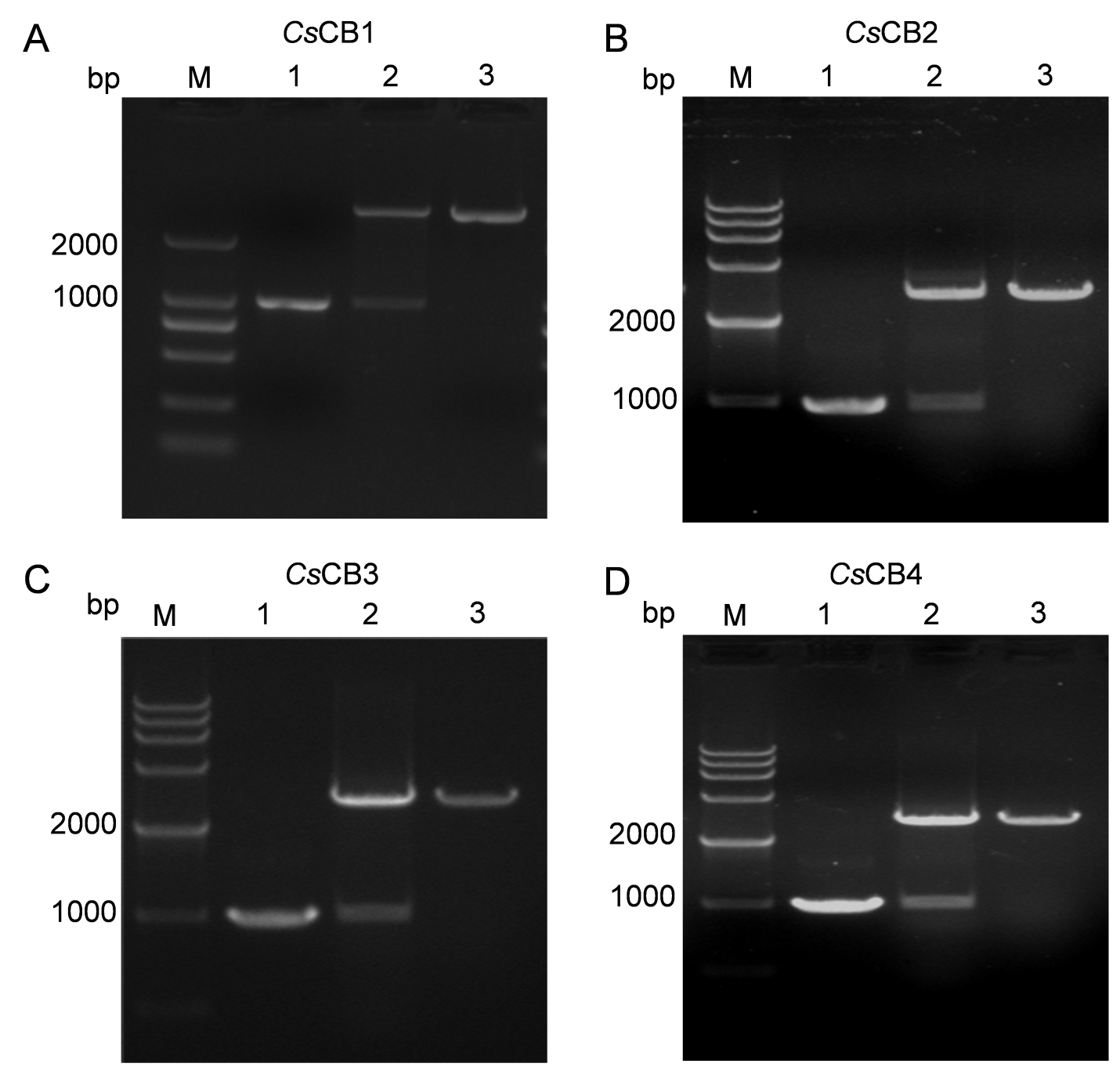


**Figure S1 Identification of recombinant plasmids by PCR amplification and restriction enzyme digestion.** M, DNA ladder. Lane 1, PCR product using recombinant plasmid as template. Lane 2, digested product from recombinant plasmid. Lane 3, digested product of empty pPICZαB. **(A-D)** *Cs*CB1, *Cs*CB2, *Cs*CB3 and *Cs*CB4, respectively. The nucleic acid products were separated by 1% agarose gel and visualized by ethidium bromide.

Figure S2


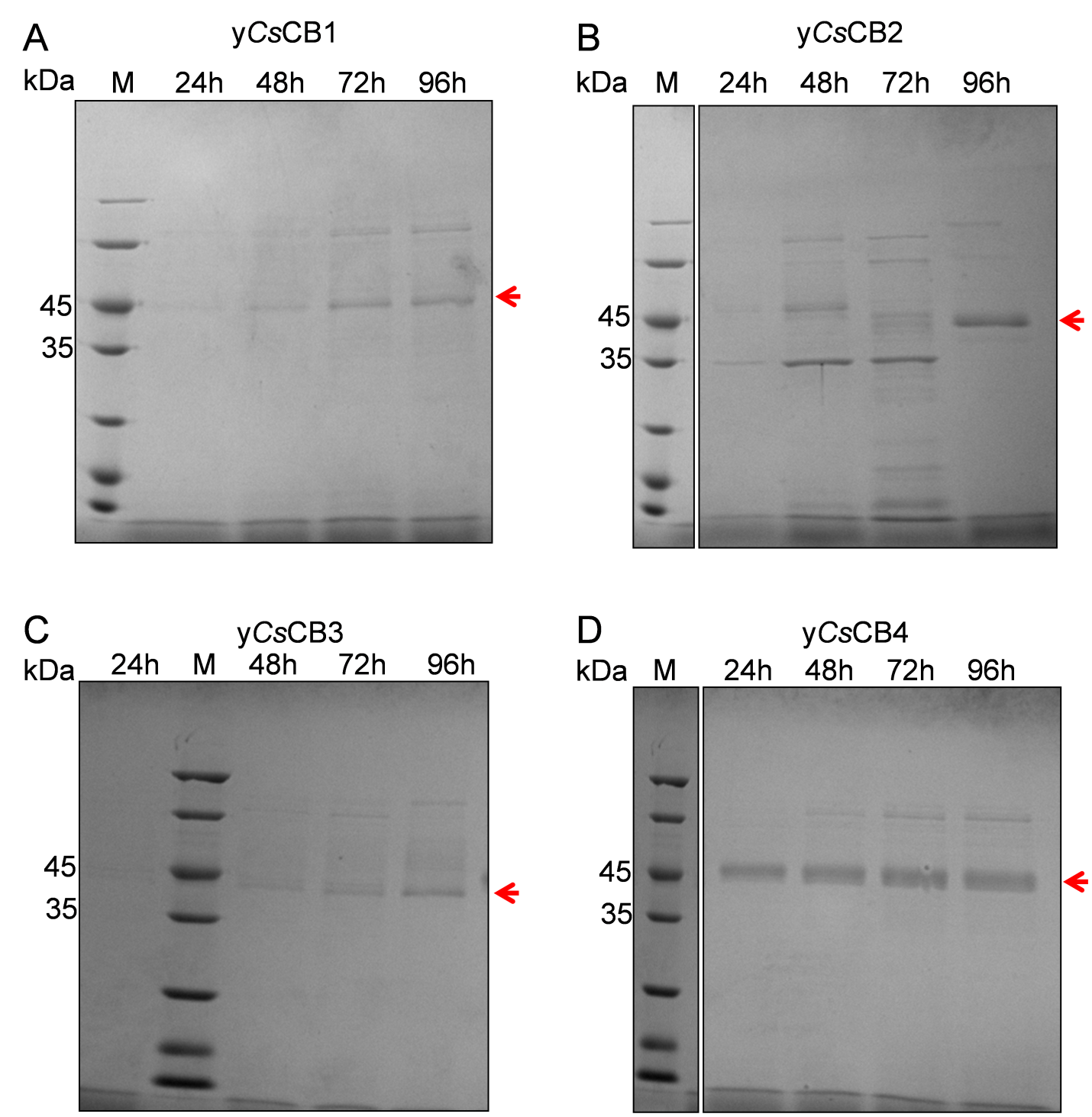


**Figure S2 Identification of extracellular expression of y*Cs*CBs by 12% SDS-PAGE.** M, protein marker. Lane 1-4 indicated supernatant samples of *Cs*CBs-pPICZaB-X33 induced by methanol for 24 h, 48 h, 72 h and 96 h. **(A-D)** y*Cs*CB1, y*Cs*CB2, y*Cs*CB3 and y*Cs*CB4, respectively. Red arrows indicated potential expression of y*Cs*CBs.

Figure S3


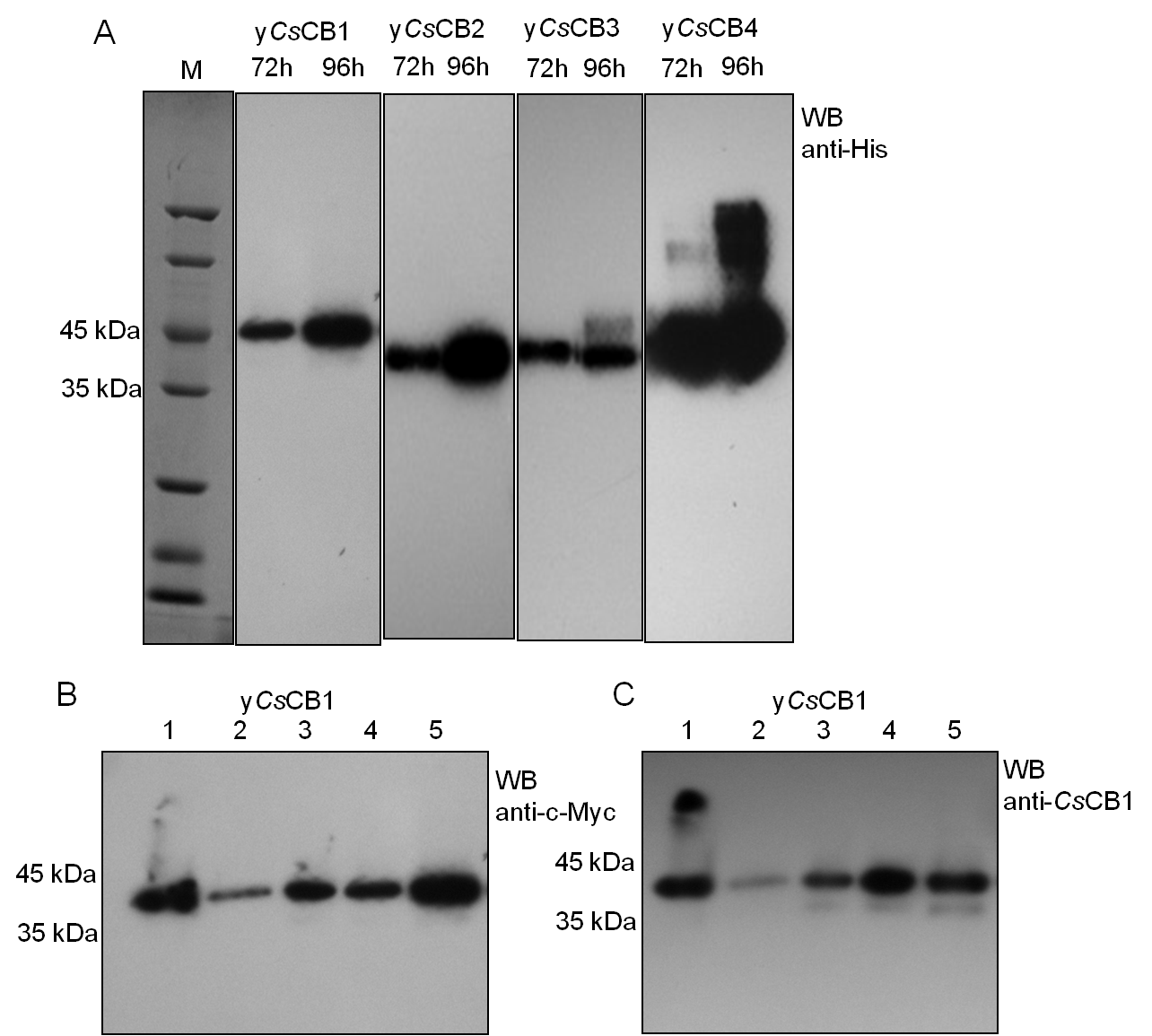


**Figure S3 Identification of extracellular expression of yCsCBs by Western Blotting.** **(A)** Cell culture medium of yCsCBs (yCsCB1, yCsCB2, yCsCB3 and yCsCB4) was probed with mouse anti-His antibody (1: 500). **(B)** Cell culture medium of yCsCB1 was probed with mouse anti-c-Myc antibody (1: 500). **(C)** Cell culture medium of yCsCB1 was probed with rat anti-CsCB1antibody (1: 800). Lanes 1-5 indicated ammonium sulfate precipitates, 72-h culture medium, 96-h culture medium, 120-h culture medium and 144-h culture medium, respectively.

Figure S4


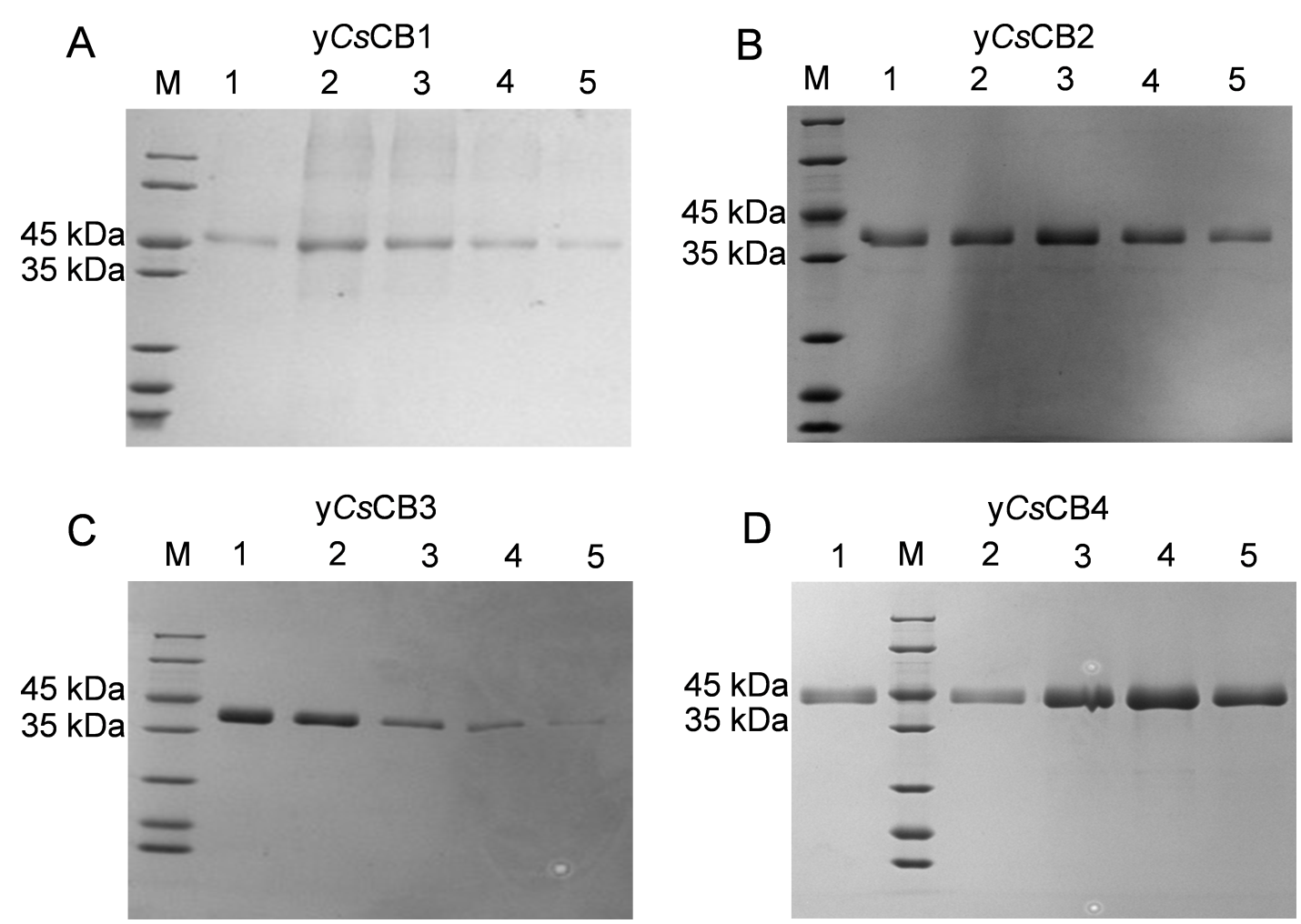


**Figure S4 Identification of purified protein of *Cs*CBs by SDS-PAGE.** M, protein marker. Lane 1-5, fraction of purified protein eluted with different concentration of imidazole (50 mM, 100 mM, 200 mM, 400 mM and 800 mM). **(A-D)** y*Cs*CB1, y*Cs*CB2, y*Cs*CB3 and y*Cs*CB4, respectively.

Figure S5


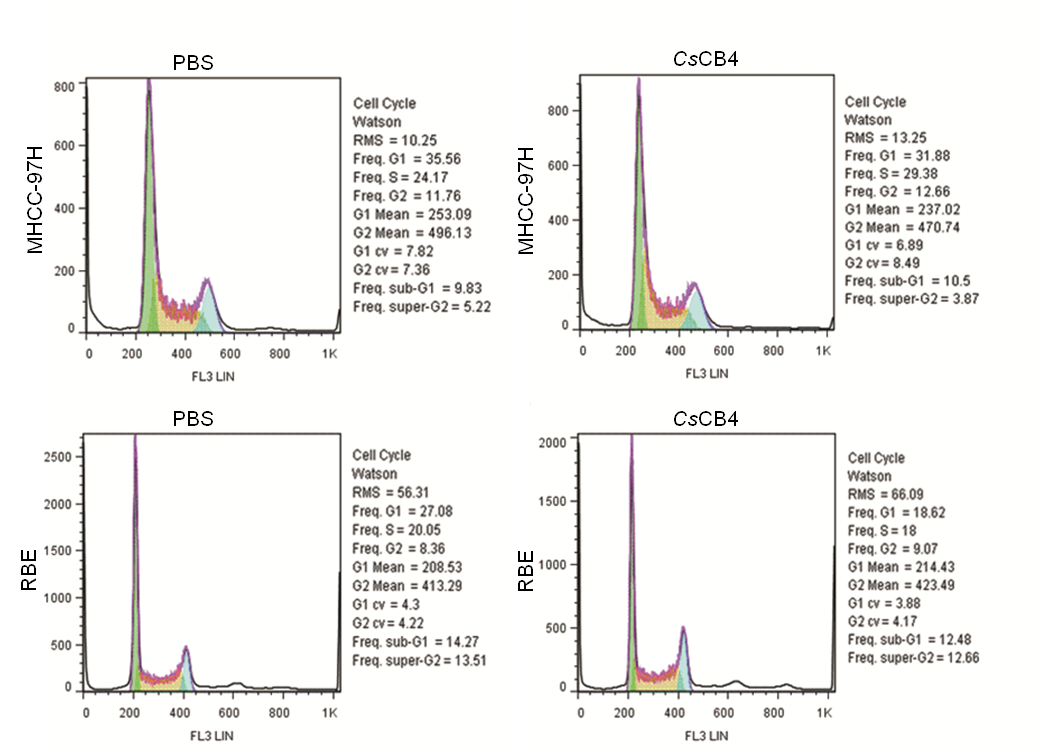


**Figure S5 Representative cell cycle analysis by flow cytometry.** MHCC-97H cells and RBE cells were incubated with PBS **(A, C)** or 1 μg/ml of *Cs*CB4 **(B, D)** for 24 h and fixed in 70% ethanol. Cell cycle distribution was determined by fluorescence activated cell sorting (FACS). The percentage of cells in G2/S period was analyzed and quantified using the FlowJo software.
